# Single anastomosis duodeno-ileal bypass with sleeve gastrectomy generates sustained improvement of glycemic control compared with sleeve gastrectomy in the diet-induced obese rat model

**DOI:** 10.1007/s13105-023-00993-x

**Published:** 2023-11-07

**Authors:** Sara Becerril, Javier A. Cienfuegos, Amaia Rodríguez, Victoria Catalán, Beatriz Ramírez, Víctor Valentí, Rafael Moncada, Xabier Unamuno, Javier Gómez-Ambrosi, Gema Frühbeck

**Affiliations:** 1 Metabolic Research Laboratory, Clínica Universidad de Navarra, Avda. Pío XII, 36, 31008 Pamplona, Spain; 2 CIBER Fisiopatología de La Obesidad y Nutrición (CIBEROBN), Instituto de Salud Carlos III, Pamplona, Spain; 3 Obesity and Adipobiology Group, Instituto de Investigación Sanitaria de Navarra, Pamplona, Spain; 4 Department of Surgery, Clínica Universidad de Navarra, 31008 Pamplona, Spain; 5 Department of Anesthesia, Clínica Universidad de Navarra, Pamplona, Spain; 6 Medical Engineering Laboratory, University of Navarra, Pamplona, Spain; 7 Department of Endocrinology and Nutrition, Clínica Universidad de Navarra, Avda. Pío XII, 36, Pamplona, Spain

**Keywords:** Sleeve gastrectomy, Single anastomosis duodeno-ileal bypass, Diet-induced obesity, Glucose metabolism

## Abstract

Bariatric surgery has become a recognized and effective procedure for treating obesity and type 2 diabetes (T2D). Our objective was to directly compare the caloric intake-independent effects of sleeve gastrectomy (SG) and single anastomosis duodenoileal bypass with SG (SADI-S) on glucose tolerance in rats with diet-induced obesity (DIO) and to elucidate the differences between bariatric surgery and caloric restriction.

A total of 120 adult male Wistar rats with DIO and insulin resistance were randomly assigned to surgical (sham operation, SG, and SADI-S) and dietary (pair-feeding the amount of food eaten by animals undergoing the SG or SADI-S surgeries) interventions. Body weight and food intake were weekly monitored, and 6 weeks after interventions, fasting plasma glucose, oral glucose and insulin tolerance tests, plasma insulin, adiponectin, GIP, GLP-1, and ghrelin levels were determined.

The body weight of SADI-S rats was significantly (*p* < 0.001) lower as compared to the sham-operated, SG, and pair-fed groups. Furthermore, SADI-S rats exhibited decreased whole body fat mass (*p* < 0.001), lower food efficiency rates (*p* < 0.001), and increased insulin sensitivity, as well as improved glucose and lipid metabolism compared to that of the SG and pair-fed rats.

SADI-S was more effective than SG, or caloric restriction, in improving glycemic control and metabolic profile, with a higher remission of insulin resistance as well as long-term weight loss.

**Supplementary Information:**

The online version contains supplementary material available at 10.1007/s13105-023-00993-x.

## Introduction

Obesity is a complex, chronic, and relapsing condition associated with a variety of comorbidities, including type 2 diabetes (T2D), hypertension, dyslipidemia, and certain cancers [1]. In 1973, Sims et al. first coined the term “diabesity” to emphasize the pathophysiologic interconnection between obesity and the development of T2D [46]. Although notable recent studies indicate that pharmacotherapy for diabetes in adults with overweight or obesity renders promising results [32], bariatric surgery is recognized as a safe, extremely effective, and permanent approach for weight loss in comparison with conventional therapies, delivering higher remission rates for T2D and obesity-related diseases [45, 51]. However, the most effective type of bariatric surgery for treating T2D is still controversial.

Different bariatric procedures are currently available, affecting caloric restriction, nutrient malabsorption, or both. Sleeve gastrectomy (SG) dramatically reduces the volume of the stomach, limiting gastric capacity, whereas in the single anastomosis duodeno-ileal bypass with SG (SADI-S), which combines restrictive and malabsorptive components, a small gastric pouch is created and a single duodenum-small intestine anastomosis is made, bypassing the duodenum and distal ileum [43]. Although interventions with a malabsorptive component are usually followed by an increased rate of metabolic improvement when compared to those based on restriction, the postoperative complications associated with the changes in gastrointestinal anatomy are usually higher [2]. SADI-S potentially solves many of these adverse effects while exhibiting a better T2D correction rate [8].

Nevertheless, the underlying mechanisms related to glucose homeostasis improvement after bariatric surgery need to be fully elucidated. Evidence points not only to a reduction in food intake and/or the malabsorption of nutrients as important contributors to the resolution of T2D since metabolic improvements appear early in the postoperative period prior to weight loss [24, 31, 54], suggesting other weight loss-independent mechanisms. Other theories postulate the neuroendocrine modulation through alterations in the gut-brain axis that robustly change after these procedures, such as the increased circulating bile acids levels [3] or the reduction in lipotoxicity and inflammatory state related to the accumulation of excess fatty acids in tissues [5].

Although we have previously observed that the SADI-S technique exerts a higher metabolically beneficial effect compared with the SG procedure due to increased white adipose tissue browning [4], few studies directly establish a detailed comparison of the SG and SADI-S procedures in detail. Moreover, given the clear beneficial effects of caloric restriction on glucose metabolism [53], the key question for the current study was to define the degree to which SADI-S and SG improve glucose homeostasis and whether this improvement exceeds the beneficial effects of weight loss achieved through caloric restriction alone. Thus, caloric restriction, SG, and SADI-S outcomes in rats subjected to diet-induced obesity (DIO) were examined and compared in order to test the hypothesis that the insulin resistance improvement observed early after bariatric surgery is due to caloric restriction-independent effects. This strategy may highlight the most relevant underlying mechanisms and molecular basis of metabolic improvement after bariatric surgery.

## Material and methods

### Experimental animals and study design

**Fig. 1:**
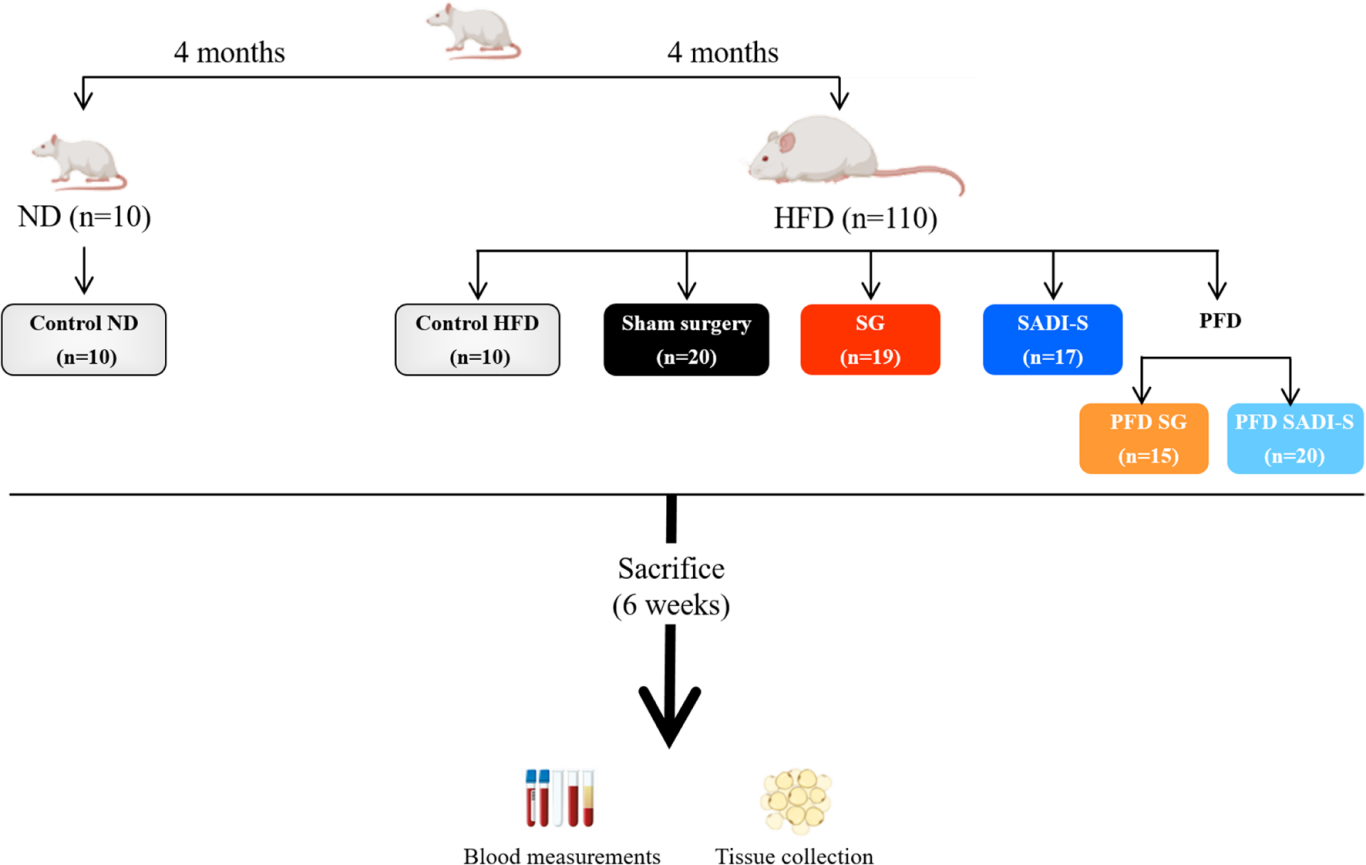
Flow diagram outlining the final experimental design. HFD, high-fat diet; ND, normal diet; PFD, pair-fed; SADI-S, single anastomosis duodeno-ileal bypass with sleeve gastrectomy; SG, sleeve gastrectomy

In total, 120 four-week-old male Wistar rats were housed singly and kept under standard lighting conditions (12-h light/dark) with a controlled temperature (22 ± 2 °C) and relative humidity (50 ± 10%) under pathogen-free conditions. Animals had ad libitum access to water, a normal diet (ND) (*n* = 10) (Diet 2014S, Harlan, Teklad Global Diets, Harlan Laboratories Inc., Barcelona, Spain), or a diet enriched with fat (HFD) (*n* = 110) (Diet F3282, Bio-Serv, Frenchtown, NJ, USA). After 4 months, rats were randomly divided into SADI-S (*n* = 24), SG (*n* = 21), or sham surgery (*n* = 20) groups. In addition to the surgical intervention groups, two further surgically naïve groups of animals with obesity were pair-fed with the same amount of food consumed by surgical rats [PFD-SADI-S (*n* = 20) and PFD-SG (*n* = 15)] in order to demonstrate that the effects are not exclusively due to a reduction in food intake. After surgery, the rodents were fed an ND, whereas a group of controls with DIO (*n* = 10) continued to be given ad libitum HFD (Fig. 1). The same surgeons conducted all surgeries. All protocols for animal use conformed to the European Guidelines for the Care and Use of Laboratory Animals (directive 2010/63/EU), and the study was reviewed and approved by the Ethical Committee for Animal Experimentation of the University of Navarra (026/19).

### Bariatric surgery procedures

Preoperative preparation and postoperative care were performed as previously described [4]. Sixty-five rats underwent a SADI-S (*n* = 24), SG (*n* = 21), or sham operations (*n* = 20) as follows: SG was performed as previously mentioned [26, 40]. Briefly, a midline incision was performed, the gastrocolic and gastrosplenic ligaments were carefully separated, and the stomach was externalized. Approximately 70% of the stomach was removed, preserving the pylorus intact and leaving a long and narrow gastric pouch.

SADI-S surgery was conducted as formerly described [4]. After the midline incision of the abdominal wall, the stomach was fully externalized, and, after the so-called SG [26, 49], the small intestine was isolated outside the abdominal cavity, and the point to be anastomosed, 35 cm proximally from the ileocecal valve, was identified. The ileum was anastomosed to the gastric pouch, bypassing the whole duodenum and distal ileum and allowing food to pass through the esophagus to the tubular gastric remnant and directly into the distal ileum.

Sham surgery involved similar incisions, operative conditions, handling of the stomach, and time without intestinal excisions. The sham group initially included animals that received either a sham-SADI-S variant or a sham-SG operation, as described before. Since the study reported no differences in the parameters measured between both sham procedures, the data are displayed as a single group. The mortality rate of SG and SADI-S animals was 10% and 29%, respectively. Body weight and food intake were routinely determined, and 6 weeks after the surgical and caloric interventions, rats were fasted for 6 h and decapitated. White adipose tissue (WAT), skeletal muscle, and liver were carefully dissected and weighed. Total white adiposity was calculated as the total sum of the different WAT deposits (epididymal, perirenal, and subcutaneous) weights. Blood samples were collected into serum separation tubes, and sera samples were obtained after centrifugation at 700 × *g* for 15 min at 4 °C and stored at − 80 °C.

### Excess weight loss and food efficiency ratio

Percentage total weight loss (%TWL) was calculated as follows: (pre-surgery body weight − final body weight) × 100 / pre-surgery body weight. The food efficiency ratio (FER) was determined for each rodent as total body weight gain per week/total food intake (kilocalories) during this period.

### Blood measurements

The glucose concentration was determined automatically by a glucometer (Ascensia Elite, Bayer, Barcelona, Spain). Serum leptin, adiponectin, insulin, and glucagon-like peptide 1 (GLP-1) were assessed using commercially available ELISA (Crystal Chem, Inc., Chicago, IL, USA) as previously reported [20, 27, 28, 39]. Intra- and inter-assay coefficients of variation were 4.6% and 5.5%, 3.5% and 6.4%, 2.6%, and 5.3%, as well as 4.2% and 7.8%, respectively. Total ghrelin and gastric inhibitory polypeptide (GIP) levels were also determined by ELISA (Millipore, Billerica, MA, USA). Intra- and interassay coefficients of variation were 0.8% and 2.8% for the former, and 3.9% and 6.0% for the latter.

Serum concentrations of free fatty acids (FFA) (Wako Chemicals, GmbH, Neuss, Germany), triacylglycerols (TG), total cholesterol (Infinity, Thermo Electron Corporation, Melbourne, Australia), and glycerol (Sigma, St. Louis, MO, USA) were quantified enzymatically using commercially available kits [10]. Hepatic TG content was measured using an enzymatic colorimetric assay, in accordance with previously published procedures [38]. Both HOMA-IR (fasting serum insulin (μU/ml) × fasting plasma glucose (mmol/L) / 22.5) and the quantitative insulin sensitivity check index (QUICKI), 1/[log(fasting insulin μU/mL) + log(fasting glucose mg/dL)] were used to quantify the degrees of insulin resistance. The adipocyte insulin resistance (Adipo-IR) index, proposed as a reliable surrogate measure to examine adipocyte dysfunction, was determined as fasting FFA (mmol/L) × fasting insulin (pmol/L).

### Oral glucose and intraperitoneal insulin tolerance tests

The oral glucose tolerance test was performed in 12-h fasted rats. Rats were administered with 50% d-glucose solution (2 g/kg of body weight) by oral gavage, and glucose concentrations were measured before (baseline) and 15, 30, 60, and 120 min after the administration. For the intraperitoneal insulin tolerance test (IPITT), a dose of 0.5 IU/kg insulin was injected intraperitoneally in conscious, 12-h fasted rats. Blood glucose was measured before (baseline) and 15, 30, 60, 90, and 120 min after the insulin injection. Glucose levels were checked by a glucometer (Ascensia Elite, Bayer, Barcelona, Spain) in the blood vein tail. The areas under the curves (AUC) of glucose levels were calculated according to the trapezoidal method.

### Statistical analysis

The data were summarized using the mean ± standard error of the mean (SEM). The test used for the assessment of normality was Kolmogorov–Smirnov’s test. Differences between groups were evaluated using the unpaired Student’s *t*-test and one-way ANOVA followed by Bonferroni's post hoc tests. The degree of association between two variables was measured using Pearson’s correlation coefficients (*r*). Statistical analysis of the data was performed with the SPSS/Windows version 15.0 software (SPSS, Inc., Chicago, IL, USA), and GraphPad Prism version 8.3 (GraphPad Software, Inc., San Diego, CA) was used to generate figures.

## Results

### SADI-S improves body weight and fat mass to a higher extent than SG in a caloric restriction-independent manner

**Fig. 2:**
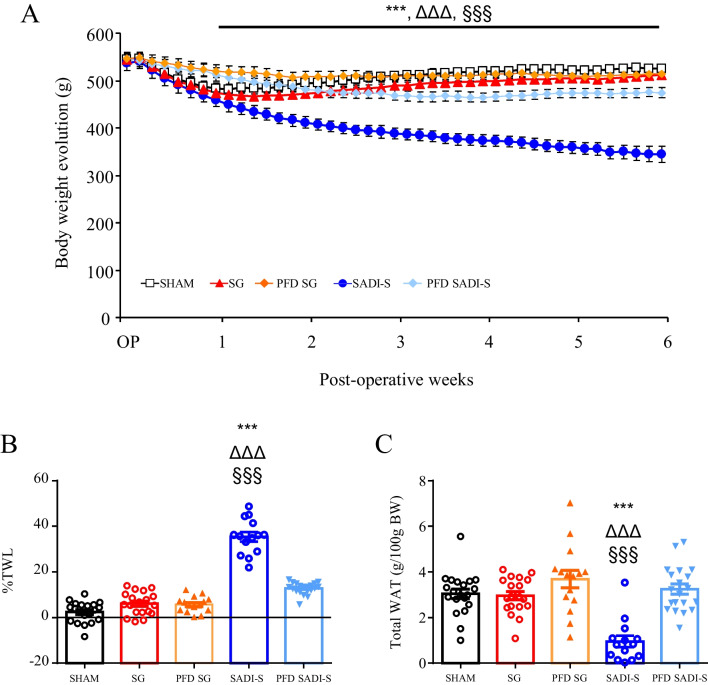
The improvement of body weight and adiposity after SADI-S, but not SG, is beyond caloric restriction. **A** Growth curves of rats 6 weeks after surgical and caloric interventions. Bar graphs illustrate **B** the total weight loss of the experimental animals and **C** the total white adipose tissue. Values are the mean ± SEM (*n* = 15–20/group). Differences were analyzed by one-way ANOVA followed by Bonferroni post hoc tests. ***p < 0.001 vs. sham-operated group; ΔΔΔ *p* < 0.001 vs. SG group; ^§§§^*p* < 0.001 vs. PFD SADI-S rats. BW, body weight; FER, food efficiency ratio; PFD, pair-fed; SADI-S, single anastomosis duodeno-ileal bypass with sleeve gastrectomy; SG, sleeve gastrectomy; TWL, total weight loss; AT, white adipose tissue

As expected, rats fed an HFD exhibited an increased body weight, total adiposity, and an impaired metabolic profile (Supplemental Table 1). As previously observed [4], SG surgery induced a rapid, although transient, body weight loss, being the maximal weight loss shown 1 week after surgery. However, the final body weight was no longer significantly different from that of sham- and age-matched rats after SG (Fig. 2A). The SADI-S group experienced the greatest weight loss, exhibiting a final body weight significantly (*p* < 0.001) lower than that of their sham- and SG-operated rats, reflected in the %TWL. Importantly, the SADI-S rats lost more weight not only than the sham-operated and SG groups, but also than their pair-fed counterparts (*p* < 0.001) (Fig. 2B). These differences were not observed between the SG rats and their pair-fed group, which steadily gained weight throughout the experimental period (Fig. 2A, B). The decreased body weight in SADI-S rats was associated with significant reductions in whole-body fat mass (*p* < 0.001), including epididymal (*p* < 0.001), subcutaneous (*p* < 0.001), and perirenal (*p* < 0.001) fat pads, as compared to SG rodents. Of note, no differences in body weight and fat mass between sham-operated groups and rats undergoing SG were observed (Table 1; Fig. 2C). Noteworthy, pair-fed SADI-S animals exhibited higher final body weight and whole adiposity and decreased %TWL (all *p* < 0.001) than SADI-S group (Fig. 2B, C), suggesting an additional effect of the SADI-S operation beyond caloric restriction. In addition, no differences were observed in SG rats regarding epididymal, subcutaneous, perirrenal, and total WAT compared with their pair-fed group.

**Table 1:** Food intake and body composition after bariatric surgery and caloric intervention in the experimental groups

Determination	Sham surgery (*n* = 20)	SG (*n* = 19)	Pair-fed SG (*n* = 15)	SADI-S (*n* = 17)	Pair-fed SADI-S (*n* = 20)	*p*-value
Final body weight (g)	526 ± 8	512 ± 8	515 ± 9	345 ± 18***^,ΔΔΔ,§§§^	475 ± 10	** < 0.001**
FI (g/day)	23.9 ± 0.4	24.5 ± 0.7	23.8 ± 0.23	17.0 ± 1.1***^,ΔΔΔ^	15.7 ± 0.4	** < 0.001**
Relative FI (kcal/day/100 g BW)	14.7 ± 0.3	15.6 ± 0.4	14.9 ± 0.32	14.9 ± 0.8^§§§^	11.2 ± 0.36	** < 0.001**
FER	0.054 ± 0.013	0.061 ± 0.023	0.015 ± 0.023	-0.279 ± 0.061***^,ΔΔΔ,§§§^	0.089 ± 0.024	** < 0.001**
Epididymal WAT (g/100 g BW)	1.14 ± 0.07	1.16 ± 0.07	1.27 ± 0.13	0.45 ± 0.11***^,ΔΔΔ,§§§^	1.12 ± 0.07	** < 0.001**
Subcutaneous WAT (g/100 g BW)	0.87 ± 0.06	0.88 ± 0.06	1.05 ± 0.11	0.32 ± 0.06***^,ΔΔΔ,§§§^	0.99 ± 0.08	** < 0.001**
Perirrenal WAT (g/100 g BW)	1.03 ± 0.11	0.96 ± 0.12	1.36 ± 0.18	0.30 ± 0.10**^,Δ,§§^	1.13 ± 0.15	** < 0.001**

Data are the mean ± SEM. Statistical differences were analyzed by one-way ANOVA followed by Tukey’s post hoc test. Bold lettering indicates statistically significant values

BW, body weight; FER, food efficiency ratio; FI, food intake; SADI-S, single anastomosis duodeno-ileal bypass with sleeve gastrectomy; SG, sleeve gastrectomy; WAT, white adipose tissue

^*^p < 0.05, **p < 0.01, and ***p < 0.001 vs. sham-operated group; ^Δ^*p* < 0.05, ^ΔΔ^*p* < 0.01, and ^ΔΔΔ^*p* < 0.001 vs. SG group; ^§^*p* < 0.05, ^§§^*p* < 0.01, and ^§§§^*p* < 0.001 vs. the corresponding pair-fed group

### SADI-S reduces food intake and improves the food efficiency ratio

Caloric intake was registered daily after the surgery. A liquid diet was given to the animals for several postsurgical days, and this period has not been taken into account in the estimations. After the reintroduction of a standard pelleted solid diet ad libitum, the daily food intake in the sham-operated group increased to 24 ± 1 g/day and remained stable until the end of the experiment. The daily food intake of both SADI-S- and SG-operated rats appeared to plateau after 2.5 weeks until the end of the experimental phase (data not shown). The absolute food intake of SADI-S-operated rats was significantly lower (*p* < 0.001) than that of sham- and SG-operated animals and correlated to a large extent with postsurgical weight loss (*r* = 0.67; *p* < 0.001). Nevertheless, no significant differences in the caloric content of the food eaten by the experimental groups were observed (Table 1). Given that the FER indicates the animal’s efficiency of feed conversion, negative values are often observed after dietary interventions or following an SG, as previously reported [19]. Interestingly, the higher %TWL observed in SADI-S animals was not associated with differences in their relative food intake but was accompanied by lower feed conversion in comparison with SG and pair-fed rats (both *p* < 0.001) (Table 1).

### SADI-S ameliorates glucose metabolism to a higher extent than SG beyond caloric restriction

**Fig. 3:**
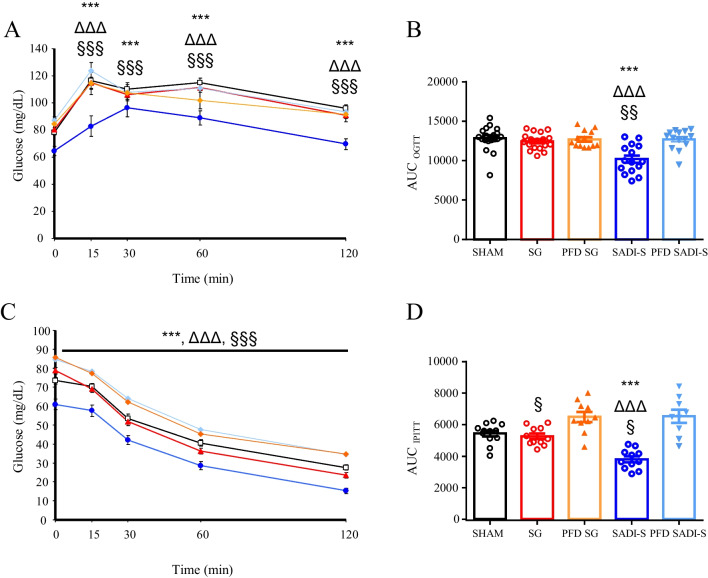
The beneficial effect of SADI, but not SG, on glycemic control is independent of caloric restriction. **A** Evolution of glucose after oral glucose tolerance and **C** intraperitoneal insulin tolerance tests. **B** Bar graphs show the AUC in the oral glucose tolerance and **D** intraperitoneal insulin tolerance tests. Values are the mean ± SEM (*n* = 15–20/group). Differences were analyzed through one-way ANOVA followed by Bonferroni post hoc tests. ***p < 0.001 vs. sham-operated group; ^ΔΔΔ^*p* < 0.001 vs. SG group; ^§^*p* < 0.05, ^§§^*p* < 0.01, ^§§§^*p* < 0.001 vs. PFD SADI-S rats. AUC, area under the curve; IPITT, intraperitoneal insulin tolerance test; OGTT, oral glucose tolerance test; PFD, pair-fed; SADI-S, single anastomosis duodeno-ileal bypass with sleeve gastrectomy; SG, sleeve gastrectomy

Since improved glycemia, insulinemia, HOMA index, and adipo-IR index were observed in animals submitted to SADI-S surgery [4] in previous studies, we focused on the comparison of glucose metabolism improvement following SG or SADI-S. The OGTT revealed an early and significant (*p* < 0.001) decrease in blood glucose levels in the SADI-S group after glucose administration in comparison with the SG group (Fig. 3A). There were no significant differences in the glucose AUC between sham- and SG-operated rats, while the glucose AUC_OGTT_ of SADI-S rodents was significantly lower than that of the sham and SG groups (*p* < 0.001 for all) (Fig. 3B). The IPITT test followed a similar pattern (Fig. 3C, D). As shown in Table 2, SADI-S was associated with decreased insulin resistance, as demonstrated by a higher QUICKI index (*p* < 0.001) as well as reduced glycemia (*p* < 0.001), insulinemia (*p* < 0.01), and HOMA index (*p* < 0.001) as compared to SG group.

**Table 2:** Food intake and body composition after bariatric surgery and caloric intervention in the experimental groups

Determination	Sham surgery (*n* = 20)	SG (*n* = 19)	Pair-fed SG (*n* = 15)	SADI-S (*n* = 17)	Pair-fed SADI-S (*n* = 20)	*p*-value
Glucose (mg/dL)	78 ± 2	80 ± 1	85 ± 4	64 ± 3***^,ΔΔΔ,§§§^	87 ± 3	** < 0.001**
Insulin (ng/mL)	4.2 ± 0.4	3.6 ± 0.3	4.2 ± 0.5	1.62 ± 0.23***^,ΔΔΔ^	2.8 ± 0.3	** < 0.001**
Adpn (µg/mL)	19.8 ± 3.0	19.8 ± 3.0	15.2 ± 2.3	13.6 ± 3.0	19.6 ± 3.5	0.434
Leptin (ng/mL)	4.6 ± 0.58	4.03 ± 0.43	5.68 ± 0.88	0.38 ± 0.06***^,ΔΔΔ,§§§^	3.26 ± 0.49	** < 0.001**
Adpn/Lep ratio	4.05 ± 0.79	4.92 ± 1.01	2.70 ± 0.41	32.23 ± 7.78***^,ΔΔΔ,§§§^	7.37 ± 1.75	** < 0.001**
Total ghrelin (ng/mL)	0.53 ± 0.06	0.42 ± 0.07	0.45 ± 0.07	2.02 ± 0.43***^,ΔΔΔ,§§§^	0.67 ± 0.10	** < 0.001**
GLP-1 (pg/mL)	6.9 ± 1.3	8.3 ± 2.0	5.8 ± 1.5	14.5 ± 3.1*^,Δ,§^	4.8 ± 1.2	**0.011**
GIP (pg/mL)	51.3 ± 8.9	36.6 ± 3.8	33.8 ± 3.9	30.8 ± 5.9	56.0 ± 8.7	0.052
HOMA	0.98 ± 0.11	0.95 ± 0.10	1.01 ± 0.12	0.31 ± 0.01***^,ΔΔΔ^	0.76 ± 0.08	** < 0.001**
QUICKI	0.40 ± 0.01	0.39 ± 0.01	0.39 ± 0.01	0.49 ± 0.01***^,ΔΔΔ^	0.41 ± 0.001	** < 0.001**
FFA (mg/dL)	18.3 ± 0.8	21.6 ± 1.0	24.1 ± 1.3	17.7 ± 1.3^§^	22.1 ± 1.4	**0.005**
Cholesterol (mg/dL)	61.1 ± 3.1	59.7 ± 3.6	61.5 ± 1.9	45.0 ± 4.4**^,Δ,§§^	65.6 ± 2.6	**0.001**
TG (mg/dL))	143 ± 13	115 ± 12	133 ± 16	95 ± 17	120 ± 15	0.071
Glycerol (mg/dL)	0.018 ± 0.002	0.018 ± 0.001	0.023 ± 0.001	0.016 ± 0.001	0.026 ± 0.001	0.920
Adipo-IR	23.8 ± 3.2	27.5 ± 3.3	27.1 ± 3.2	8.7 ± 1.8*^,ΔΔ^	15.9 ± 3.8	** < 0.001**
Liver (g)	13.2 ± 0.4	12.5 ± 0.4	12.3 ± 0.3	7.7 ± 0.3***^,ΔΔΔ,§§§^	10.4 ± 0.3	** < 0.001**
Liver (g/100 g BW)	2.59 ± 0.05	2.45 ± 0.07	2.37 ± 0.05	2.45 ± 0.07^§§^	2.25 ± 0.03	** < 0.001**
Intrahepatic TG (mg/g liver)	5.0 ± 0.7	4.5 ± 1.0	2.8 ± 0.2	2.3 ± 0.8*	2.5 ± 0.6	**0.032**

Values presented as the mean ± SEM. Differences between groups were analyzed by two-way ANOVA followed by Tukey’s post hoc test. Bold lettering indicates statistically significant values

Adipo-IR, adipose tissue insulin resistance index; Adpn, adiponectin; FFA, free fatty acids; GIP, gastric inhibitory polypeptide; GLP-1, glucagon-like peptide 1; HOMA, homeostasis model assessment; QUICKI, quantitative insulin sensitivity check index; SADI-S, single anastomosis duodeno-ileal bypass with sleeve gastrectomy; SG, sleeve gastrectomy; TG, triglycerides

^*^p < 0.05, **p < 0.01, and ***p < 0.001 vs. sham-operated group; ^Δ^*p* < 0.05, ^ΔΔ^*p* < 0.01, and ^ΔΔΔ^*p* < 0.001 vs. SG group; ^§^*p* < 0.05, ^§§^*p* < 0.01, and ^§§§^*p* < 0.001 vs. the corresponding pair-fed group

SADI-S-induced increase in insulin sensitivity was also noticed in comparison with pair-fed animals, and these differences were not observed between SG-operated rats and their corresponding pair-fed animals (Table 2). Taken together, our data suggest that the anti-diabetic effects of SADI-S are not exclusively a consequence of the reduced caloric intake.

### Differential effect of SADI-S and SG on the profile of hormones regulating appetite and glucose metabolism

To further explore the metabolic differences following SADI-S and SG procedures and their association with caloric restriction, changes in the circulating concentrations of representative factors regulating food intake, adipose tissue function, and glucose metabolism were analyzed (Table 2). As expected, the absence of obesity in SADI-S rats was also indicated by the reduced plasma leptin levels compared to sham-operated and SG animals (*p* < 0.001 for all). Importantly, the suggested biomarker of dysfunctional adipose tissue Adpn/Lep ratio [20] significantly augmented (*p* < 0.001) after weight loss in rats submitted to SADI-S in comparison with sham and SG groups (Table 2). GLP-1 plasma levels were also increased after SADI-S surgery (*p* < 0.05 vs. sham, *p* < 0.05 vs. SG, *p* < 0.05 vs. pair-fed group). Intriguingly, SADI-S rats showed a strong increase in circulating total ghrelin (*p* < 0.001) compared with their counterpart sham or SG animals, according to previous studies [4]. A tendency toward decreased levels of GIP was also observed, despite not reaching statistical significance (*p* < 0.10). These differences were maintained under caloric restriction (Table 2). A tendency toward a negative correlation between GLP-1 and AUC_OGTT_ (*r*: − 0.33; *p* = 0.080) and AUC_IPITT_ (*r*: − 0.35; *p* = 0.060) was found.

### Lipid profile changes after SADI-S and SG depend on caloric restriction

Circulating fasting plasma levels of FFA did not differ among SADI-S and sham groups, whereas significant differences in plasma TG and total cholesterol levels (both *p* < 0.01) were observed (Table 2). Plasma levels of TG, total cholesterol, and FFA were significantly reduced (*p* < 0.05 all) in the SADI-S group compared to the SG group, and these differences disappeared when compared to pair-fed rats (Table 2), indicating that the SADI-S effects on the lipid profile are exclusively due to the food intake reduction.

SADI-S reduced the hepatic weight as well as steatosis (*p* < 0.001 for each), but these differences were not observed in sleeve-gastrectomized rats (Table 2). While pair-feeding to SADI-S was also associated with a reduction in liver weight as well as intrahepatic TG (both *p* < 0.05), these differences were not shown in the pair-fed to SG rodents (Table 2).

## Discussion

Bariatric surgery is considered an efficient treatment for obesity, showing long-term results regarding weight loss and obesity-related improvements, including T2D remission [32]. Roux-en-Y gastric bypass as well as SG are currently the most widely used bariatric procedures applied in the treatment of obesity and T2D [30]. However, the mechanisms underlying the remarkable bariatric surgery-induced effects on body weight and glycemic control remain unclear, and the remission of T2D observed seems to be partly due to weight-loss-independent mechanisms [33]. The anatomical and physiological changes after SADI-S and SG are very different, but both surgeries are effective, to varying degrees, in achieving weight loss and T2D remission [16, 44]. In this context and considering that our previous work showed an improvement in glucose homeostasis [4], the objectives of the present study were (1) to assess and compare the effect of both bariatric surgeries on glucose metabolism in a rodent model of obesity and (2) to differentiate the metabolic effects induced by bariatric surgery that are independent of caloric restriction.

Whereas SG rats displayed a constant weight regain analogous to the sham-operated controls shortly after the surgery without differences in food intake, the present study confirms that SADI-S surgery induces rapid, safe, and sustained body weight loss, mainly attributable to a reduction in adiposity. This post-surgical weight loss exhibited a strong correlation with reduced absolute food intake, without differences in relative caloric intake. The body composition improvement may be related to the increased SCWAT browning verified in previous studies by our group [4]. Noteworthy, SADI-S pair-fed rats showed increased body weight despite their reduced food intake.

SADI-S surgery was also related to a higher remission rate of insulin resistance than SG at 6 weeks after surgery, as evidenced by lower glycemia, insulinemia, HOMA index, and increased QUICKI index compared to SG rats. In this sense, the greater reduction of total fat pads observed in the SADI-S group should be considered a key component for remission of this pathology. Strikingly, the improvements in glycemic control cannot be correlated exclusively to the decreased food intake. In order to delve into the underlying mechanism that leads to a postsurgical increase in insulin sensitivity, an analysis of circulating levels of traditional and novel gastrointestinal hormones involved in the regulation of glucose metabolism was performed. The total levels of ghrelin, an orexigenic hormone secreted from the gastric fundus and duodenal enteroendocrine cells, are decreased in obesity, insulin resistance, and T2D [47]. Given that the major part of the stomach is either removed or bypassed after SG and SADI-S procedures, ghrelin levels would be expected to decrease postoperatively. In the present research work, it was observed that, despite the resection of the stomach in both surgeries, after the SADI-S intervention, not only the circulating levels of ghrelin were maintained but also increased. However, the effect of bariatric procedures on ghrelin levels remains controversial. Procedures such as SADI-S have not yet been studied enough, and no ghrelin results have been published so far [48]. The relevance of the increased ghrelin levels remains in question since ghrelin-deficient mice exhibit the same degree of body weight loss and improvements in glucose tolerance in response to SG [11]. We clearly observed that circulating levels of ghrelin were unaffected by SG but increased with the SADI-S surgery as weight loss continued to decrease, suggesting that the postsurgical ghrelin levels are not directly involved in the metabolic improvements after SADI-S. These results are in contrast with earlier studies performed by Cummings et al. in diabetic rats [13]. The reasons for the different effects of SG are not clear. One of the plausible reasons may be that Cummings et al. performed their experiments in the University of California Davis-T2D rat model, characterized by the development of adult-onset polygenic obesity, insulin resistance, and hyperglycemia, whereas the present study was performed in Wistar rats, prone to developing obesity in response to an HFD. Furthermore, differences may be attributed to a distinct study design: the animals of the former study were followed until at least 5 months after surgery, whereas the rats in the present study were killed 6 weeks postsurgery. Finally, it is important to note that changes in the surgeons or little modifications in the surgical technique may modify the results of the experiment. Multiple factors, rather than a single one, are responsible for weight control and metabolic outcomes.

There are two main gut hormones identified as incretins: GLP-1 and GIP. GLP-1 is produced and secreted by intestinal enteroendocrine L cells found mainly in the ileum, while GIP is secreted by the enteroendocrine K cells that are present mainly in the duodenum and upper jejunum [7, 9]. Both GLP-1 and GIP are secreted after food ingestion, and they enhance insulin secretion in a glucose-dependent manner. Several studies have shown an increase [6, 50] in fasting GLP-1 levels after malabsorptive surgeries, while no increase after a restrictive bariatric procedure was observed [37]. In the present study, SADI-S significantly increased GLP-1 levels, likely contributing to the greater improvement in glucose tolerance. Moreover, the slight reduction of the glucagonotropic agent GIP after bariatric surgery may also be responsible for improving diabetes, as proposed by Rubino et al. [41], although the impact of bariatric surgery on GIP is highly controversial. Probably, GIP secretion may depend on the alimentary and common limb length of the bariatric surgery; the shorter the bypass of the proximal jejunum, the greater the GIP response. This intricate interplay of factors in the entero-insular axis seems to play a key role in improving the insulin response, thus improving T2D. The impact of other factors cannot be discarded [42]. Furthermore, the improvement in glucose metabolism was not observed in pair-fed animals, suggesting that the negative calorie balance does not constitute the predominant mechanism underlying the early metabolic changes after bariatric surgery.

Moreover, obesity can increase cardiovascular morbidity and mortality through direct or indirect effects, including insulin resistance, hypertension, and dyslipidemia [23]. A review described that bariatric surgeries yield beneficial effects on the remission of dyslipidemia and cardiovascular incidence [12]. In this sense, the purpose of the study was also focused on the comparison of both surgeries in terms of enhancement of lipid homeostasis in rats with DIO. We have observed a significant decrease in serum lipid levels after SADI-S surgery in comparison with SG. SADI-S improves the lipid profile by decreasing plasma TG, total cholesterol, and FFA. SG did not affect fasting levels of TG, liver weight, or hepatic lipid storage, whereas SADI-S rats also exhibited decreased liver weight as well as intrahepatic TG, contributing to the decreased plasma lipids observed. These improvements were not observed in pair-fed rats, being related to changes beyond a simple reduction in caloric intake that extend to the well-known effects of adipokines on the regulation of lipolysis and lipogenesis [17–19, 21, 34, 35]. Furthermore, the involvement of other factors also participating in body weight, lipid, and glucose metabolism control with aquaporins cannot be discarded [15].

One of the strengths of the study was that the animal model used constitutes a critical tool for exploring the physiological mechanisms underlying both bariatric surgeries and permits the detailed study of the factors that contribute to weight loss in reproducible conditions. Moreover, the large sample size of the different experimental groups provided accurate mean values, and animals were killed for a long period of time after surgery (6 weeks), allowing the study of the post-bariatric physiological mechanisms involved in the metabolic improvement. Nevertheless, the present work has several limitations that deserve consideration. One limitation of our study is that, although we have analyzed the incretin hormones GLP-1 and GIP, we have not included other important gastrointestinal hormones related to the beneficial metabolic effects of bariatric surgery. It is well documented that bariatric surgery leads to profound changes in the secretion of gut hormones, with an important impact on insulin secretion, appetite, and satiety. In this regard, Le Roux et al. published data from both animal and human studies showing a high release of not only GLP-1 but also the L-cell product peptide-YY (PYY), which is considered an appetite-regulating hormone [25]. Moreover, bariatric surgery increases the secretion of cholecystokinin or oxyntomodulin, secreted by the L and I cells in the gastrointestinal mucosa, respectively, with an important role in appetite regulation [52]. Neurotensin, secreted from the gastrointestinal endocrine cells and related to accelerated gastric emptying, or the gastric hormone gastrin, implicated in glycemic control, is also related to the mechanisms behind the positive effects of bariatric surgery [14, 29]. Recent in-depth analyses have shown that incretin hormones are most biologically relevant when they are elevated in the postprandial state. In this sense, fasting plasma GLP-1 concentrations were substantially increased in the SADI-S rat group (likely due to chronic stimulation of gastrointestinal L-cells and L-cell hypertrophy), whereas fasting plasma GIP levels were unaffected. Regrettably, there are still continued disparities about the effectiveness and sustainability of the improvement of T2D in patients with obesity, since most evaluations of bariatric surgical outcomes have been hampered by inadequate and/or incomplete long-term follow-up [36]. Finally, the study has been performed only in male rats, so it prevents a correct evaluation of how bariatric surgery interacts with sex. Additional studies would be required to elucidate the potential influence of sex on surgical outcomes. It has to take into account that data should be properly studied for their transferability to human physiology, and the anatomical differences between rats and humans need to be considered.

Our current findings showed continued durability of glycemic improvement after SADI-S, persistent weight loss, and reductions in glycemia not observed following SG or caloric restriction. In summary, SADI-S has proved to be more efficacious concerning weight loss and metabolic comorbidity resolution when compared with SG and caloric restriction. We highlight that SADI-S in the rat is a suitable model to study the metabolic consequences of shortening the intestinal tract. However, further studies evaluating the impact of SADI-S surgery on energy homeostasis in animal models and humans will facilitate a better understanding of the metabolic changes and mechanisms following this bariatric surgery technique. A thorough knowledge of these effects could help optimize surgery techniques to provide the maximum antidiabetic impact.

### Supplementary Information

Below is the link to the electronic supplementary material.

Supplementary file1 (DOCX 25.8 KB)

## Data Availability

The datasets used and/or analyzed during the current study are available from the corresponding author on reasonable request.

## Declarations

### Approval of the research protocol

N/A.

### Approval date of registry and the registration No. of the study/trial

N/A.

### Animal studies

All protocols for animal use conformed to the European Guidelines for the Care and Use of Laboratory Animals (directive 2010/63/EU), and the study was approved by the Ethical Committee for Animal Experimentation of the University of Navarra (026/19).
