# Supplementary material for: Single anastomosis duodeno-ileal bypass with sleeve gastrectomy generates sustained improvement of glycemic control compared with sleeve gastrectomy in the diet-induced obese rat model
Source: J Physiol Biochem. 2023 Nov 7;80(1):149–60. doi: 10.1007/s13105-023-00993-x (PMC10810039; doi:10.1007/s13105-023-00993-x)
Supplement: Supplementary file 1 — Supplementary file1 (DOCX 25.8 KB) [file 13105_2023_993_MOESM1_ESM.docx]

# Supplemental Table 1. Body weight, adiposity and metabolic profile in control groups.

| Determination | | Normal diet  (n=10) | High-fat diet  (n=10) | | *P* | | |
| --- | --- | --- | --- | --- | --- | --- | --- |
| Body weight (g) | | 512 ± 8 | 544 ± 17 | | **0.006** | | |
| Epididymal WAT (g/100g BW) | | 1.11 ± 0.07 | 1.64 ± 0.12 | | **<0.001** | | |
| Subcutaneous WAT(g/100g BW) | | 0.96 ± 0.06 | 1.36 ± 0.11 | | **0.002** | | |
| Perirrenal WAT (g/100g BW) | | 0.81 ± 0.12 | 1.56 ± 0.21 | | **0.004** | | |
| BAT (g/100g BW) | | 0.12 ± 0.01 | 0.15 ± 0.01 | | 0.463 | | |
| Total WAT (g/100g BW) | | 2.87 ± 0.20 | 4.56 ± 0.37 | | **0.021** | | |
| Food intake (g) | | 22.1 ± 0.4 | 11.9 ± 1.6 | | **<0.001** | | |
| Relative food intake (kcal/day/100 g BW) | | 14.0 ± 0.2 | 14.7 ± 0.5 | | 0.151 | | |
| FER | | 0.043 ± 0.010 | 0.10 ± 0.02 | | **0.016** | | |
| Rectal temperature (ºC) | | 36.1 ± 0.1 | 37.1 ± 0.0 | | **0.003** | | |
| Glucose (mg/dL) | 73 ± 5 | | | 87 ± 5 | | **0.005** |  |
| Insulin (ng/mL) | 3.82 ± 0.52 | | | 6.09 ± 0.95 | | **0.049** |  |
| HOMA | 0.77 ± 0.12 | | | 1.30 ± 0.17 | | **0.020** |  |
| QUICKI | 0.41 ± 0.01 | | | 0.37 ± 0.01 | | **0.019** |  |
| AUC OGTT | 13449 ± 399 | | | 15452 ± 532 | | **0.006** |  |
| AUC IPITT | 5873 ± 358 | | | 8692 ± 502 | | **<0.001** |  |
| FFA (mg/dL) | 18.0 ± 1.2 | | | 18.8 ± 1.1 | | 0.627 |  |
| TG (mg/dL) | 153 ± 11 | | | 182 ± 14 | | 0.114 |  |
| Cholesterol (mg/dL) | 60 ± 4 | | | 71 ± 3 | | **0.046** |  |
| Glycerol (mg/dL) | 0.023 ± 0.001 | | | 0.027 ± 0.002 | | 0.235 |  |
| Adipo-IR index | 19.9 ± 3.4 | | | 35.1 ± 6.9 | | 0.059 |  |
| Leptin (ng/mL) | 5.3 ± 0.6 | | | 9.8 ± 1.6 | | **0.015** |  |
| Adiponectin (μg/mL) | 15.0 ± 2.8 | | | 8.8 ± 3.9 | | 0.051 |  |
| Adpn/leptin ratio | 0.47 ± 0.08 | | | 0.16 ± 0.09 | | **0.021** |  |
| Liver (g) | 12.7 ± 0.3 | | | 13.2 ± 0.5 | | 0.060 |  |
| Liver (g/100g BW) | 2.50 ± 0.04 | | | 2.47 ± 0.04 | | 0.080 |  |
| Intrahepatic TG (mg/g liver) | 9.6 ± 3 | | | 26.9 ± 7.6 | | **0.040** |  |
| Total ghrelin (ng/mL) | 0.53 ± 0.07 | | | 0.40 ± 0.06 | | 0.138 |  |
| GLP-1 (ng/mL) | 5.9 ± 1.1 | | | 7.1 ± 1.3 | | 0.500 |  |

# Adpn, adiponectin; AUC, area under the curve; BAT, brown adipose tissue; BW, body weight; FER, food efficiency ratio; FFA, free fatty acids; GLP-1, glucagon-like peptide-1; HOMA, homeostasis model assessment; IPITT, intraperitoneal insulin tolerance test; OGTT, oral glucose tolerance test; QUICKI, quantitative insulin sensitivity check index; TG, triglycerides; WAT, white adipose tissue. Values presented as the mean ± S.E.M. Statistical differences were analyzed by the Student’s *t* test.
